# “Have You Taken the A4 Challenge?” Correlates and Impact of a Thin Ideal Expression From Chinese Social Media

**DOI:** 10.3389/fpsyg.2021.669014

**Published:** 2021-06-07

**Authors:** Todd Jackson, Xiaoxuan Ye, Brian J. Hall, Hong Chen

**Affiliations:** ^1^Department of Psychology, University of Macau, Taipa, China; ^2^School of Psychology, Southwest University, Chongqing, China; ^3^School of Global Public Health, New York University Shanghai, Shanghai, China

**Keywords:** thin feminine ideal, Chinese, social media, culture, gender, disordered eating

## Abstract

In three studies, we assessed knowledge, correlates, and effects of the A4 challenge, an expression of the thin ideal from Chinese social media. In Study 1, gender differences in familiarity with the A4 challenge were assessed among 225 women and 151 men. Compared to men, women and female peers from participant social networks were more familiar with and likely to have taken the challenge themselves. In Study 2, body image experiences of women who passed the A4 challenge (*N* = 45) and average weight peers who did not pass the challenge (*N* = 75) were assessed. The former group reported fewer weight concerns and less social pressure to lose weight but no group differences were observed with respect to binge-eating, dieting, or other compensatory weight loss behaviors. As such, eating disorder symptoms did not account for the experience of passing the A4 challenge. In Study 3, changes in state body dissatisfaction were assessed among 205 women randomly assigned to view images of (1) thin peers successfully passing the challenge vs. (2) thin or (3) average size controls. The absence of condition differences in post-exposure state body dissatisfaction indicated exposure to A4 challenge portrayals *per se* did not cause increases in negative appearance self-evaluations for women in general. However, among women who were exposed to A4 challenge images, but not control group women exposure to other images, trait body dissatisfaction predicted increased post-exposure state dissatisfaction, independent of pre-exposure state dissatisfaction. Implications are discussed in relation to effects of exposure to the A4 challenge and conceptualizing the task as a “challenge.”

## Introduction

Perceived pressure to be thin and internalization of the thin feminine attractiveness ideal from traditional mass media and social media have been linked to body dissatisfaction and eating disorder symptoms in questionnaire studies of girls and young women in China (e.g., Chen and Jackson, 2012; Jackson and Chen, 2014, 2015). Whereas, some authors have speculated that Westernization and thin ideal depictions from Western media account for body dissatisfaction and problem eating behaviors among young Chinese women (e.g., Jung, 2018; Zhang et al., 2018; Rodgers et al., 2020), the impact of thin ideal portrayals in Chinese media has been even more pronounced based upon self-report data from cross-sectional (Jackson et al., 2016) and longitudinal (Jackson et al., 2020) study designs.

Despite such evidence, to date, implications of particular thin ideal depictions from Chinese social media have not evaluated in either correlational studies or experimental research. Hence, it is not clear if or how experiences with such imagery are related to or change body image in Chinese samples. Given that reviews of the experimental literature have found exposure tends to have weak overall causal effects on outcomes (Ferguson, 2013; Hausenblas et al., 2013), conclusions about the salience of thin ideal internalization for body image based on subjective reports may not extend to experiments assessing the impact of exposure to thin ideal stimuli. In this research, questionnaire and experimental methods were adopted to examine correlates and causal effects of contact with the “A4 challenge,” an expression of the thin body ideal that emerged recently from Chinese social media.

**Figure 1:**
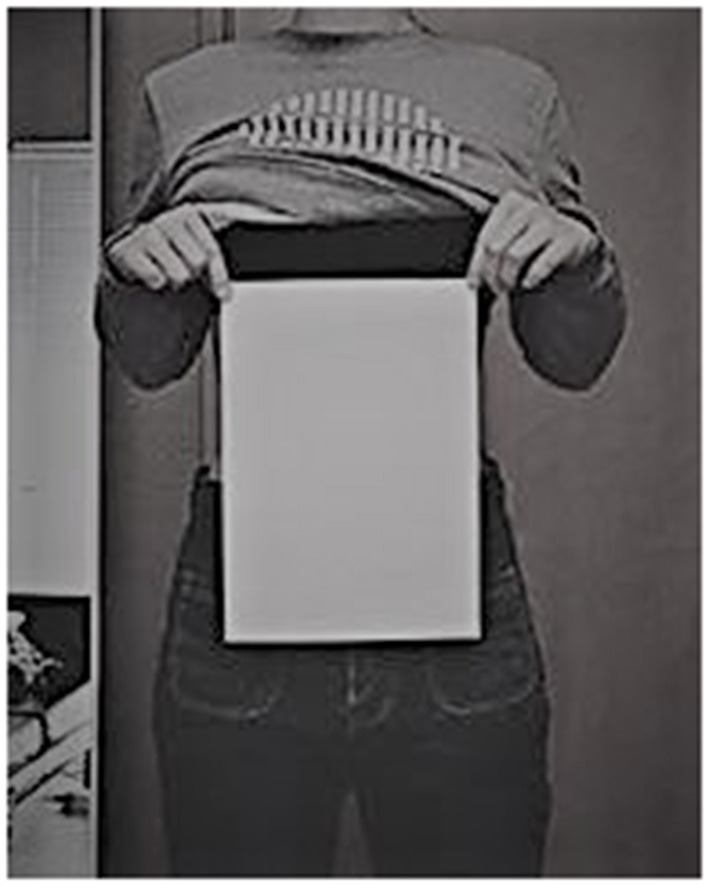
Illustration of woman taking and passing the A4 challenge.

Origins of the A4 challenge can be traced to an advertisement for tea seed oil, an edible oil used in cooking, that appeared in Chinese social media during early 2016. The ad featured a young woman passing the A4 challenge, which involves demonstrating that one's waist can be completely hidden by the 8.27″ width of a standard, letter-sized A4 sheet used in Europe and Asia (see Figure 1), presumably due to using this product. The ad promoted the idea that passing the challenge is a sign of good health but its focus on a narrow waist is consistent with thinness as a central facet of the feminine attractiveness ideal in China (e.g., Leung et al., 2001; Wu et al., 2020). Thereafter, popular young Chinese actresses and models began posting images of themselves passing the challenge on social media sites. Awareness of the task and debate about its implications spread in the general population.

Specific questions follow from this phenomenon. Given its nature, does the A4 challenge target and appeal to women in particular relative to men? Compared with average size peers, do young Chinese women who “pass” the challenge report more satisfaction with their appearance or is passing the challenge related to negative body image experiences and elevations in eating disorder symptoms? Does exposure to A4 challenge depictions cause increased state body dissatisfaction among young women? These questions were foci of three studies presented below.

## Study 1

Despite the absence of past research on gender differences in A4 challenge knowledge and attitudes, indirect evidence based on gender differences in thin ideal preferences provides a basis from hypotheses. First, based on the initial advertisement and its portrayals in Chinese social media (i.e., images of women passing the challenge), the A4 challenge appears to target Chinese women in particular. Second, given that having a slender waist is a requirement of passing, the A4 challenge aligns with emphasis on thinness as the ideal body type among women in China. Illustrating this ideal, Zhang et al. (2018) assessed weight preferences of more than 2,000 undergraduate Chinese women; about 73% of those surveyed reported taking action to lose weight during the past 6 months and another 57% expressed a desire to be “much thinner” than at present even though just over 2% had a body mass index (BMI) in overweight or obese ranges. Finally, the higher prevalence of fatness concerns/eating disorders (e.g., Chen et al., 2006; Chen and Jackson, 2008; Jackson and Chen, 2010) and status of thin ideal internalization as a risk factor for later increases in eating disorder symptoms (Jackson and Chen, 2008a) among young Chinese women also underscore thinness as a key feature of the Chinese feminine ideal. Given such data, we hypothesized that young Chinese women and female peers would be more aware of and more likely to have taken the A4 challenge compared to young Chinese men and male peers.

### Materials and Methods

#### Participants and Procedure

The sample comprised 376 undergraduate students (225 women, 151 men) recruited from a large Chinese university. Participants ranged in age from 18 to 29 years (*M* = 20.27, *SD* = 1.50), with a majority (73%) in their first or second year of undergraduate study. On average, respondents had a BMI of 20.51 (*SD* = 2.72).

After receiving approval from the Human Research Ethics Committee of the university during June 2016, volunteers were solicited via online advertisements for a study on attitudes toward social media portrayals of appearance. Those who expressed interest completed a brief questionnaire during scheduled times in a classroom setting. The questionnaire included an informed consent and a series of forced-choice or open-ended items related to demographics and knowledge of/attitudes toward the A4 challenge. Time needed to complete the research was 10–15 min on average. Participants received eight Chinese yuan (~1.20 USD) for participation.

#### Measures

##### A4 Challenge Survey

The first two authors created a brief survey that assessed basic demographics (gender, age, year of study, height in cm, weight in kg) and an item querying whether one has heard of the A4 challenge (no vs. yes). For those answering “yes” to this item, follow-up items assessed (i) how long ago they first heard of the A4 challenge (in weeks), (ii) where they had first heard of the challenge (“social media” vs. “traditional media” vs. “other”) and (iii) accuracy of understanding via an open-ended query, “Please provide a correct description of the A4 challenge in the space below.” Descriptions were coded independently as accurate vs. inaccurate by two psychology graduate students who assisted in data collection. With three exceptions, raters agreed on all descriptions. Disagreements were resolved in consultation with the first author.

Participants were also asked if they had personally taken the A4 challenge (no vs. yes) and to provide open-ended estimates of numbers of female and male peers who had (i) discussed the challenge with them and (ii) taken the challenge. Participants were asked whether they thought the A4 challenge was useful as a simple index of overall health (“*No* = *1,” “Neutral/Undecided* = *2,” “Yes* = *3”*). Finally, participants were asked to rate how attractive they thought people who pass the A4 challenge are on a nine-point likert scale with “*1* = *Very Unattractive,” “5* = *Neither Unattractive or Attractive,”* and “*9* = *Very Attractive”* as defined anchors or mid-points.

#### Design and Data Analysis

Gender differences on nominal-level categorical measures and ordinal measures of A4 challenge items were tested with chi-square tests and independent samples *t*-tests, respectively. Paired samples *t*-tests were run to assess sample differences between female and male peers reported to have discussed and taken the A4 challenge. Effect sizes for 2 × 2 and 2 × 3 chi-square tests were psi and Cramer's V coefficients, respectively; Cohen's d was the effect size statistic used for *t*-tests. For each of these statistics, effect sizes of 0.20, 0.50, and 0.80 were interpreted as small, medium, and large. Finally, bivariate correlation analyses assessed within-gender relations of BMI with A4 knowledge and attitudes. G^*^Power 3.0 indicated the desired group size for each gender was at least 102 participants for independent samples *t*-tests using an alpha of 0.05, a power of 0.80, and an effect size of 0.35.

### Results

In general, the A4 challenge campaign was familiar to the sample, as 325 participants (86.4%) said they knew what the challenge entails and 295 (78.5%) provided accurate descriptions of the task. As hypothesized, more women than men were aware of the challenge and described it in an accurate way (Table 1). Moreover, as predicted, within the subgroup that provided accurate descriptions, women were significantly more likely than men to report having heard about the task on social media, to have personally taken the A4 challenge, and to have reported knowing more female peers who had taken the challenge, though no gender difference was found for estimated number of male peers reported to have taken the challenge (Table 2). Finally, as hypothesized, more female than male peers were reported to have (1) discussed the challenge (female peers: *M* = 3.27, *SD* = 4.55 vs. male peers: *M* = 0.96, *SD* = 1.76), *t*_(295)_ = 9.35, *p* < 0.001, *d* = 0.59 and (2) taken it (female peers: *M* = 1.13, *SD* = 2.11 vs. male peers: *M* = 0.09, *SD* = 0.42), *t*_(295)_ = 8.69, *p* < 0.001, *d* = 0.68.

**Table 1:** Gender differences on categorical measures of A4 challenge knowledge, experience, and perceptions (*N* = 376).

**Measure**	**Women**	**Men**			
	***N* (%)**	***N* (%)**	***χ^2^***	***p***	**Φ/*V***
**Knowledge of A4 challenge**					
No	19 (8.4%)	32 (21.2%)	12.52	0.001	0.18
Yes	206 (91.6%)	119 (78.8%)			
**Accurate description of A4 challenge^a^**					
No	37 (16.4%)	44 (29.1%)	8.62	0.003	0.15
Yes	188 (83.6%)	107 (70.9%)			
**Source of learning about A4 challenge**					
Social media	137 (72.9%)	63 (58.9%)	6.79	0.033	0.15
Traditional media	14 (7.4%)	9 (8.4%)			
Other (e.g., friends)	37 (19.7%)	35 (32.7%)			
**Have you taken the A4 challenge?**					
No	160 (85.1%)	101 (94.4%)	5.77	0.016	0.14
Yes	28 (14.9%)	6 (5.6%)			

a *After this point, results are based on those who gave correct A4 challenge descriptions (n = 295)*.

**Table 2:** Gender differences in personal awareness of A4 challenge, peer group experiences of A4 challenge, and perceptions of A4 challenge as an index of health and attractiveness (*N* = 295).

**Measure**	**Women (*n* = 188)**	**Men (*n* = 107)**			
	***M* (*SD*)**	***M* (*SD*)**	***t***	***p***	***d***
Time you have been aware of A4 challenge (weeks)	7.94 (4.42)	6.97 (4.50)	1.78	0.077	0.21
Total female peers who have discussed A4 challenge	4.23 (5.15)	1.60 (2.51)	5.89	0.001	0.69
Total female peers who have taken A4 challenge	1.91 (1.79)	1.07 (1.74)	2.09	0.038	0.24
Total male peers who have discussed A4 challenge	0.51 (1.20)	1.75 (2.25)	5.29	0.001	0.62
Total male peers who have taken A4 challenge	0.07 (0.34)	0.13 (0.53)	0.99	0.266	0.12
A4 challenge as a general health index	2.35 (0.70)	2.14 (0.78)	2.25	0.026	0.28
A4 challenge as an attractiveness index	5.48 (1.52)	5.43 (1.87)	0.24	0.814	0.03

Other Study 1 analyses were exploratory in nature. For example, within the subgroup that generated accurate A4 challenge descriptions, both women and men reported more same sex than other sex peers had discussed the challenge with them. Furthermore, those who pass the challenge were rated as moderately attractive, on average (*M* = 5.46, *SD* = 1.65), though there was no significant gender difference in attractiveness ratings (Table 2).

Finally, regarding within-gender associations of BMI with A4 knowledge and attitudes, women with lower BMIs were more likely to report awareness of the A4 challenge (*r* = −0.16, *p* = 0.019). Among women who provided accurate challenge descriptions, lower BMIs were related to having taken the challenge oneself (*r* = −0.31, *p* < 0.001) and knowing more other women who had done so (*r* = −0.18, *p* = 0.014). BMI was not related to women's perception of the challenge as a useful index for one's health status (*r* = −0.081, *p* = 0.267) or attractiveness ratings of those who pass the challenge (*r* = 0.01, *p* = 0.929), though positive perceptions of the challenge as useful health status index correlated with higher attractiveness ratings of those who pass the challenge, *r* = 0.41, *p* < 0.001. For men, correlations between BMI and other measures were not significant (all *p*'s > 0.09), though positive views of the challenge as a useful health status index correlated with attractiveness ratings of those who passed the challenge, *r* = 0.35, *p* < 0.001.

### Discussion

Together, Study 1 results indicated the A4 challenge was more salient to Chinese women than men. Women were more likely than men to be aware of and accurately describe the A4 challenge. Among participants who provided accurate descriptions, women were more likely than men to have personally taken the challenge and report female peers who had discussed and taken the challenge. These effect sizes were small, perhaps because substantial majorities within each gender were aware of the challenge but only small minorities had actually taken the challenge. Nonetheless, complementing past China-based research (e.g., Leung et al., 2001; Zhang et al., 2018), the pattern of gender differences in experience with the A4 challenge provides further support for the centrality of thinness in Chinese conceptions of the feminine attractiveness ideal.

Within-gender analyses indicated the A4 challenge is especially relevant for women with lower BMIs; compared to their heavier peers, these women were more aware of and more likely to have taken the challenge themselves and reported more women in their social networks had also done so. Select laboratory research has found women with higher BMIs and/or increased body dissatisfaction levels display more rapid attention disengagement from (e.g., Gao et al., 2011) and weaker attention biases toward (e.g., Glauert et al., 2010) thinner bodies than women with lower BMIs and/or less body dissatisfaction do. Tentatively, the former group may be more susceptible to increases in negative affect upon being exposed to thin ideal portrayals. In response, they may avoid or divert attention from such depictions to protect self-esteem and reduce discomfort or anxiety induced by comparing their appearance to that of thinner women. While body satisfaction was not assessed in this sample, women who had lower BMIs may have been more receptive to information about the A4 challenge because it was less threatening to their self-image.

Finally, on average, women held a more positive view than men did of using the A4 challenge as an index of one's general health. Past work has found Chinese women have stronger preferences than Chinese men do for thinness as a feature of the feminine appearance ideal (Chen et al., 2006). Unfortunately, it was not clear if positive views of the task as a measure of health status reflected these lines of evidence. An open-ended follow-up item soliciting *reasons* for positive vs. negative appraisals of using the challenge as a general health index might have aided in elucidating this finding.

Nonetheless, appraisals of the A4 challenge as an index of health vs. attractiveness were not independent based on highly significant, positive within-gender correlations between these measures. On one hand, risk for cardiovascular-related disease and mortality in Asian samples has been linked to lower waist circumference (WC) or BMI cut-off values than those of other racial groups (e.g., Li et al., 2002; Koster et al., 2008; Zhang et al., 2009). However, such data are often based on comparisons of first or second vs. fourth or fifth quintiles in WC or BMI distributions. Therefore, higher risk tends to reflect more extreme WC and BMI values than very low vs. average comparisons. At best, the A4 challenge is only a crude proxy for WC measurement that is unlikely to apply to most people in lower risk groups. Equating good health with passing the A4 challenge is also troubling in light of evidence that lower BMI and small waist circumference increases risk for certain illnesses (e.g., hip fractures, lung cancer) and mortality in some subgroups of women, independent of smoking status (e.g., Folsom et al., 2000; Dolan et al., 2007) as well as serious medical consequences of unhealthy weight loss behaviors that can accompany the pursuit of ultra-thinness (e.g., Mitchell and Crow, 2010).

Does passing the A4 challenge reflect an unhealthy pursuit of ultra-thinness among young Chinese women? Assessing differences in appearance perceptions and behaviors of women who pass the challenge vs. average size peers might clarify how membership in these subgroups relates to presence of or risk for problem eating behaviors. This issue was the focus of Study 2.

## Study 2

Sociocultural models have posited that average weight or overweight women who experience a gap between their own physical appearance and the thin attractiveness ideal are susceptible to disturbances in body image and engage in disordered eating behaviors as a means of reducing appearance social pressure and discrepancies between their own appearance and the ideal (e.g., Stice, 2001; Thompson and Stice, 2001; Knauss et al., 2008). Although self vs. ideal discrepancies have been assessed in general samples via thin ideal internalization scales featuring items such as “I want to be thinner” and “I want to look like women in fashion magazines,” the nature of ideals is only implicit in such studies. In experiments featuring explicit depictions of the thin attractiveness ideal, control condition stimuli can be problematic. For example, in studies examining exposure to thin models vs. household objects, it is not clear whether effects are due to thinness or humanness.

As an alternative approach to assessing correlates of self vs. thin ideal gaps, comparisons can be made between average weight women who do not pass the A4 challenge and peers who pass the A4 challenge and more closely approximate the thin ideal. On one hand, support for certain premises of sociocultural accounts would be found if women who pass the A4 challenge experience more body satisfaction and less appearance social pressure than do average size women. Conversely, if disordered eating is a means of attaining the ideal, then women who pass the A4 challenge should report more problematic eating and weight/shape management.

Toward clarifying these issues, experiences of body image and disordered eating were assessed among women who passed the A4 challenge vs. women with an average BMI who did not pass the challenge. Because members of the former group had smaller discrepancies between their own body size and the ideal, we hypothesized that weight concerns, sociocultural influences on appearance (i.e., pressure, comparisons), and eating disorder symptoms would be more attenuated among women who pass the A4 challenge.

### Materials and Methods

#### Participants and Procedure

Participants were 120 women recruited. The sample ranged in age from 18 to 25 years of age (*M* = 20.78, *SD* = 1.83) and had a mean BMI of 19.35 (*SD* = 1.91). Given the study focus on comparisons of women who pass the A4 challenge vs. average weight women who do not pass the challenge, data from four other women were excluded because their BMI exceeded the average range cutoff (18.5–23) for Chinese samples (e.g., Li et al., 2002).

After receiving ethics approval from the participating university's Human Research Ethics Committee, volunteers were recruited through a post on the campus electronic bulletin board seeking (1) women who had recently passed the A4 challenge and (2) average weight women for a questionnaire study on attitudes and behavior related to one's physical self. Members of the former group were also asked to submit an image of themselves passing the A4 challenge and members of the latter group answered “no” to a screening item querying whether they could pass the A4 challenge. About 1–3 weeks later, volunteers completed the survey in a small classroom setting. Upon arriving at their scheduled appointments, the women read and signed an informed consent that described research requirements (i.e., completion of brief questionnaires), time involved (20–30 min), and compensation (20 yuan).

Questionnaire completion followed. English-language scales had previously undergone Mandarin translation to English back-translation from bilingual Ph.D. students. All measures had been validated previously in samples of young Chinese women, including factor structures that replicated original scale structures, except where noted below (Chen et al., 2006; Jackson and Chen, 2008b, 2011, 2015; Chen and Jackson, 2012). Furthermore, all scale internal consistencies were good (i.e., between α = 0.80 and α = 0.89) in the current sample). Higher scale scores always reflected more frequent or intense experiences. Upon handing in their surveys, the women were debriefed and paid in another room. In addition, 20 women from each subgroup were asked, at random, if they would provide 2–4 digital photos of themselves toward creating an image set for use in future studies. Volunteers were photographed (see Study 3 for details) and paid an additional 20 yuan.

#### Measures

##### Negative Physical Self Scale-Fatness Concerns (NPS-F; Chen et al., 2006)

The Chinese-language NPS includes an 11-item Fatness Concerns subscale that assess thoughts, feelings, projections, and behaviors related to fatness and overweight. Items were rated from “*1* = *Not like me at all”* to “*5* = *Very much like me.”* The NPS-F alpha in this study was α = 0.89.

##### Asian Appearance Media Pressure Scale-Weight (AAMPS-W; Jackson et al., 2016)

Paralleling NPS subscale content, the four-item AAMPS-W assessed pressure “felt from internet sites, TV, movies, social media, videos and/or magazines from China, Hong Kong, Korea and other Asian countries” related to concerns with weight. Items were rated from 0 (*No pressure at all*) to 4 (*A great deal of pressure*). The scale alpha was α = 0.87.

##### Perceived Sociocultural Pressure Scale (PSPS; Stice, 2001)

Four interpersonal PSPS items assessed perceived pressure to change one's physical appearance from friends and family. Two dating partner items were excluded since some women were not currently dating. Item response options ranged from “*1* = *Not at all”* to “*5* = *Very much.”* These items were found to load together on an “interpersonal pressure factor” and correlate with other eating disorder risk factors in past research with Chinese samples (Jackson and Chen, 2011). In this sample, the interpersonal PSPS alpha was α = 0.86.

##### Physical Appearance Comparison Scale-Revised (PACS-R; e.g., Jackson and Chen, 2008b)

Four PACS-R items assessed tendencies to make physical appearance comparison with peers. Items were rated between “*1* = *Never”* and “*5* = *Very often*. PAC-R factor structure and validity have been supported in Chinese samples (Jackson and Chen, 2008a,b; Jackson and Chen, 2011). The PACS-R alpha in the sample was α = 0.80.

##### Eating Disorder Diagnostic Scale (EDDS; Stice et al., 2000)

The 22-item EDDS is a self-report screen based on DSM-IV eating disorder criteria. The first 18 EDDS items include symptoms that are common in persons with eating disorders and non-clinical samples (e.g., eating more than usual, dieting, weight concerns) and can be standardized and summed to provide a composite measure of disordered eating. This composite has satisfactory psychometrics among American women (Stice et al., 2000) as well as a unidimensional structure, acceptable internal consistencies and convergent validity support in samples of Chinese women (e.g., Jackson and Chen, 2011, 2014). The EDDS composite alpha was α = 0.83 in this study.

##### Demographics

Age, height and weight were assessed on the basis of self-reports.

#### Design and Data Analysis

Preliminary analyses included *t*-tests to assess differences between women who pass the A4 challenge and average weight controls on age and BMI. Main analyses included a multivariate analysis of variance (MANOVA) to assess group differences on the following dependent measures (DVs): fatness concerns, weight-related appearance pressure from Chinese/Asian media, interpersonal appearance pressure from family and friends, appearance comparisons with peers and eating disorder symptoms. Assuming there was a significant multivariate effect, univariate analysis of variance (ANOVA) results were examined. Because DVs directly or indirectly reflected weight perceptions, a multivariate analysis of covariance (MANCOVA), was also run, controlling for a probable between-groups difference in BMI, to examine whether A4 challenge vs. average weight subgroup differences from the initial MANOVA were due to weight status. Power analysis for a MANOVA with two levels and five DVs was conducted in G^*^Power using an alpha of 0.05, a power of 0.80, and an effect size of 0.35. Based on these parameters, the desired sample size was estimated to be 112 participants.

### Results

#### Preliminary Analyses

There was no age difference between groups (see Table 3). However, as expected, A4 challenge group women had a lower mean BMI than their average weight peers did (Table 3). The effect size of the mean difference was large.

**Table 3:** Body image experience differences between women who pass the A4 challenge and average weight women (*N* = 120).

	**Participant group**			
	**A4 challenge (*n* = 45)**	**Average size control (*n* = 75)**		
**Measure**	**Mean (*SD*)**	**Mean (*SD*)**	***F***	**Partial Eta^**2**^**
Age (years)	20.98 (2.01)	20.65 (1.71)	0.88	0.01
Body mass index	17.43 (1.14)	20.50 (1.23)	186.48^**^	0.61
Fatness Concerns (NPS)	18.69 (6.21)	27.35 (8.43)	42.92^**^	0.27
Media pressure—weight	3.58 (3.96)	6.76 (3.39)	21.65^**^	0.16
Interpersonal appearance pressure	7.82 (2.23)	9.05 (3.31)	4.88^*^	0.04
Peer appearance comparisons	12.20 (2.63)	11.64 (3.53)	0.36	0.01
Eating disorder symptoms (EDDS)^a^	−3.43 (6.76)	2.17 (9.29)	12.38^**^	0.10

* *p < 0.05;*

** *p < 0.001*.

a *Negative standardized EDDS composite scores reflect fewer eating disorder symptoms*.

#### Main Analyses

The initial MANOVA revealed an overall group difference on the DVs, *F*_(5, 114)_ = 4.37, *p* < 0.001. Table 3 presents descriptive statistics and univariate F-values for each measure. As hypothesized, A4 challenge group women had comparatively lower mean levels of fatness concerns, weight-related pressure from Chinese/Asian media, and interpersonal appearance pressure as well as fewer total eating disorder symptoms. However, no group differences were found for peer appearance comparisons (Table 3). The follow-up MANCOVA indicated the overall group difference on DVs was no longer significant after controlling for the group difference in BMI, *F*_(5, 113)_ = 4.37, *p* < 0.221. Furthermore, univariate effects from Table 3 were no longer significant (all *p's* > 0.10) when the group difference in BMI was controlled.

#### Supplementary Analyses

Although A4 challenge group women reported significantly lower mean EDDS composite scores, preceding analyses did not shed light on the specific nature of these differences. Hence, a supplementary MANOVA was run on the 18 individual EDDS items to assess the pattern of eating disorder diagnostic criteria that differentiated groups. Despite the underpowered sample size which was originally calculated on the basis of five rather than 18 DVs, a significant multivariate effect was found, *F*_(18, 100)_ = 2.03, *p* = 0.014. Hence, univariate effects were examined. Average weight women were more likely to feel fat (*p* < 0.001), fear weight gain (*p* < 0.001), view weight as an influence on self-perceptions (*p* = 0.011), report self-recriminations after eating large amounts (*p* < 0.001), and feel upset about weight gain due to overeating (*p* = 0.002). In contrast, no group differences were found on binge-eating items (i.e., eating uncommonly large amounts, feeling out of control during such episodes, eating when not hungry, eating until uncomfortably full) or compensatory behaviors (i.e., dieting, purging, excessive exercise, laxative use). Finally, for EDDS items with skewed distributions, univariate analyses based on normalized item scores fully replicated the initial pattern of differences.

### Discussion

Study 2 indicated women who passed the A4 challenge reported fewer concerns with fatness, less pressure from Chinese/Asian media to lose weight, less pressure from family and friends to alter physical appearance, and fewer total eating disorder symptoms than average weight peers did. However, underscoring the role of weight status on effects, group differences were no longer significant when the impact of BMI differences was co-varied. Together, these findings align with select sociocultural model premises suggesting that wider gaps between current appearance and the thin ideal are related to increased appearance pressure and body image concerns (Stice, 2001; Thompson and Stice, 2001). Such accounts have been tested more extensively among Western women who tend to have higher mean BMI ranges, though overweight women were not included in this research. As such, Study 2 results highlighted how mild disturbances in weight and eating extend to average weight Chinese women relative to thin peers. Results converge with evidence that a majority of young Chinese women want to be thinner (Zhang et al., 2018) but also illustrated how average weight Chinese women experience comparatively more weight- and appearance-related pressure from social environments and overall eating disorder symptoms than their thinner peers do.

Supplementary analyses of EDDS items indicated average weight women expressed more concerns with weight and negative affect due to overeating but did not endorse more disordered eating *behaviors* related to binge-eating and compensation. Perhaps the exclusion of overweight or obese women attenuated group differences in use of binge-eating and compensatory behaviors as responses to negative weight perceptions (Jacobi, 2005). In any event, the slender body size and lower BMI of women who passed the A4 challenge did not correspond with more frequent dieting, less binge-eating, or greater use of other compensatory behaviors related to weight loss attempts. Broader implications of this finding are revisited below.

In sum, Study 2 women who passed the A4 challenge experienced fewer fatness concerns, less appearance social pressure, and lower levels of eating disorder symptoms than average weight women did. These effects could be explained by group differences in BMI. Moreover, passing the A4 challenge did not reflect less binge eating and/or more frequent compensatory behaviors. Regardless, the link between increased weight concerns and larger discrepancies with the thin ideal illustrated in Study 2 suggested that A4 challenge portrayals might cause negative appearance self-evaluations among women exposed to them. Study 3 tested this conjecture.

## Study 3

To date, experiments examining effects of contact with the A4 challenge have not been conducted. However, reviews on the impact of exposure to appearance ideals on body satisfaction provide foundations for relevant hypotheses (e.g., Ferguson, 2013; Hausenblas et al., 2013). Ferguson (2013) conducted a meta-analysis on over 200 correlational and experimental studies and concluded media exposure has a negligible overall impact on women's body satisfaction (*r* = 0.07), though the mean effect size was stronger (*r* = 0.26) among body dissatisfied women. Similarly, Hausenblas et al. (2013) examined experimental studies that included pre- and post-exposure assessments and found exposure to idealized appearance images was related to a trivial, non-significant overall increase in body dissatisfaction (*d* = 0.03). However, whereas subgroups at risk for an eating disorder displayed moderate mean increases in post-exposure body dissatisfaction (*d* = 0.34), general samples showed slight decreases (*d* = −0.03).

Small mean effects of exposure to idealized images may occur, in part, because general samples comprise an amalgam of persons with lower and higher levels of trait body dissatisfaction. Exposure might contribute to further increases in state body dissatisfaction among trait body dissatisfied women but have complementary effects on trait satisfied women whose comparisons with idealized portrayals are presumably more favorable. Indeed, Frederick et al. (2017) have noted exposure to thin ideal images can have positive effects on a subset of women (e.g., serve as a reminder that one's own body resembles the lauded ideal). As well, women who reject appearance-based definitions of self-concept may be less susceptible to negative reactions during exposure to thin ideal images. Consequently, adverse effects of viewing such imagery may be masked and average post-exposure body dissatisfaction scores of experimental conditions come to approximate control group means. In light of heterogeneity in trait body dissatisfaction levels and/or reactions to thin ideal portrayals within general samples, between-conditions assessments of mean differences can be supplemented by within condition analyses of relations between trait body dissatisfaction and changes in state body dissatisfaction following exposure to idealized images vs. control images.

From this selective overview of experimental studies, we hypothesized that young women exposed to images of thin peers passing the A4 challenge would report significantly more post-exposure state body dissatisfaction than would peers who viewed control images of thin women or average size women that did not call attention to body size, though the magnitude of this difference would be modest. In addition, the impact of trait body dissatisfaction on pre- to post-exposure changes in state body dissatisfaction was expected to be stronger among young women exposed to A4 challenge images than young women in control conditions. Given effects of BMI on weight concerns in Study 2, we also explored within condition associations between individual differences in BMI and changes in pre-test to post-exposure state body dissatisfaction.

### Materials and Methods

#### Participants and Procedure

Participants (*N* = 205) were undergraduate women ranging in age from 18 to 25 years (*M* = 20.12, *SD* = 1.61). A minority (28.3%) reported a current dating relationship whose average duration was 16.31 months (*SD* = 14.67). The sample had a mean BMI of 20.39 (*SD* = 2.41).

Procedures for gaining ethics approval paralleled those from Studies 1 and 2. Recruitment was undertaken via an online advertisement but participation was limited to individually-tested undergraduate women. After arriving at their scheduled appointments, volunteers read and signed an informed consent that outlined the research focus (i.e., studies on factors related to self-perceptions), requirements (i.e., completion of brief questionnaires, performance of a computer-based tasks), the time involved (i.e., 25–30 min), incentives (20 yuan), and the voluntary nature of participation. After signing the consent, volunteers were asked to complete three different studies; we were interested in linking responses from initial and final tasks but framed the research in terms of discrete studies or tasks to reduce demand characteristics (see Want, 2014). In the initial study, women completed a “state reactions” questionnaire featuring body dissatisfaction items and fillers related to current perceptions of personality, academic ability, and interpersonal relations followed by trait measures of body dissatisfaction and affect described below. Alpha coefficients for all questionnaires used in Study 3 were also in the “good” range. Participants were then asked to guess the main hypothesis of the questionnaire they had just completed and told they would now be doing a different study. This instruction was given as a way of attempting to reduce the likelihood that participants would link certain responses they made on the initial questionnaire (i.e., those related to state body satisfaction) to subsequent tasks related to viewing images of women and completing state body satisfaction items a second time.

The next study was a standardized vigilance task that served as a “filler” to further reduce the likelihood that responses from the previous study and final study would be linked. The women were to focus on a central fixation on a computer screen [1,000 ms] and indicate, as quickly as possible, when a yellow square appeared at random locations within 10 s of the fixation offset. Reaction time performance feedback was given on each trial. One hundred trials were run to limit the task to 5 min. Upon completion, participants were to guess the study hypothesis.

Finally, based on a priori random assignment, the women viewed one of three computer-based presentations featuring images of (1) 10 young women successfully passing the A4 challenge vs. (2) the same 10 women holding an A4 sheet and a pen up in front of their chest (i.e., “implicit” thin condition) vs. (3) 10 average size young women holding an A4 sheet and pen up in front of their chest (control condition). Holding the pen and paper up was done to imply images and comments about passing the test reflected an *academic exam* not the A4 challenge where the sheet was held in a manner that highlighted having a thin waist. In each condition, six images of young men were inserted in a fixed order as fillers because actual media sources, such as fashion magazines feature thin ideal images that are typically juxtaposed with unrelated content and accompanied by text (Want, 2014). Hence, images included cartoon bubbles with standardized Mandarin comments about passing a test or competition (e.g., “I'm glad I was able to pass this test,” “Passing the test was easier than I thought”) as if the protagonist was speaking to the viewer. Captions accompanying female images were applicable to passing the A4 challenge or passing a written exam and were the same across all conditions.

The sequence of female and male filler images and image presentation durations (10 s each) was standardized with E-Prime. The only factors manipulated between conditions were the women in portrayals (very thin vs. average size) and/or their gestures (performing A4 challenge vs. holding sheet with pen in hand). To assess and encourage attention toward images, the first and third male filler images were followed by four captions, numbered from 1 to 4. Participants were to identify which caption accompanied the preceding image by pressing the corresponding number key. Incorrect responses were followed by a prompt requesting careful attention to images and repeated presentations of the filler image until a correct response was given. A new image followed the correct answer. Following their presentation, the women completed state body dissatisfaction items embedded within the state reactions measure a second time. Next, they guessed the hypothesis of Study 3. Following the experiment, debriefing was undertaken to explain the main focus, ensure participants experienced no distress from the experiment, and answer participant queries.

#### Image Sets

**Figure 2:**
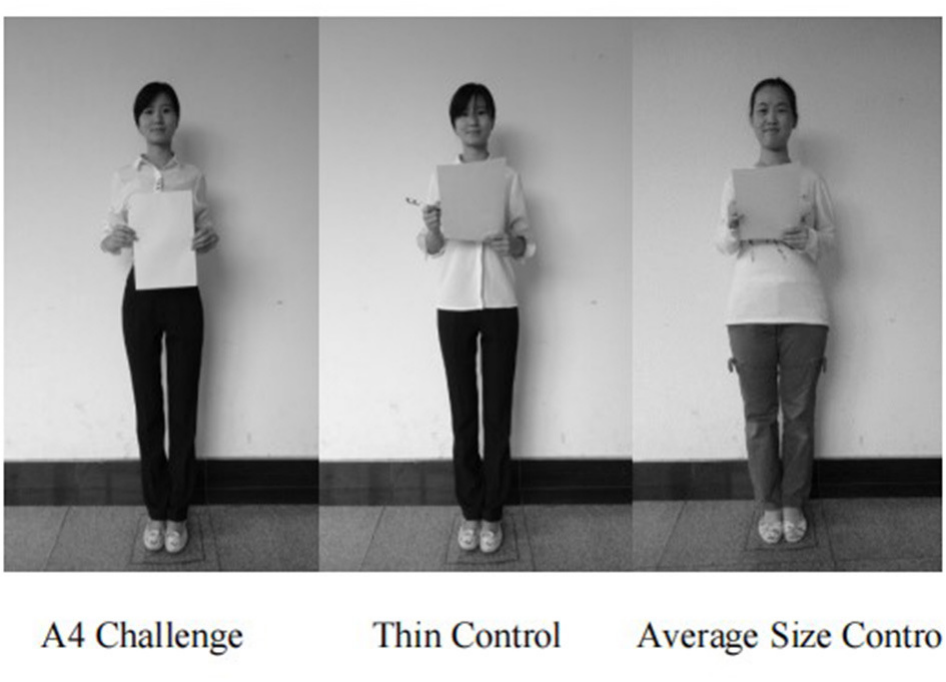
Sample images for each condition of thin ideal exposure experiment.

Digital photos of 10 thin women and 10 average size women recruited for Study 2 were used as stimuli. All volunteers were asked to stand against a white wall facing the camera with a smile. Each thinner woman was asked to pose (1) passing the A4 challenge and (2) holding an A4 sheet and pen as if having received exam feedback (thin control condition) (see Figure 2). Average size women were also photographed holding an A4 sheet and pen (average size control condition). Subsequently, cartoon bubbles with accompanying comments were super-imposed on each photo. Photos of six male graduate students standing against the white wall and holding a badminton racket were fillers. Images were transformed from color to black and white and resized to 55 × 56 mm with 100 pixels per inch.

#### Measures

##### State Body Dissatisfaction Scale

Following similar research (Ata et al., 2013), participants were asked to evaluate personal satisfaction, at this moment, with their (1) overall physical appearance, (2) body weight, (3) body size and (4) body shape from 0 = *extremely satisfied* to 5 = *extremely dissatisfied*. Responses were summed to attain total scores. As described above, items were embedded in a larger state reactions scale that included 14 filler items. State body dissatisfaction alphas were α = 0.87 and α = 0.90 for pre-exposure and post-exposure phases.

##### Satisfaction and Dissatisfaction With Body Parts Scale (SDBPS; Stice, 2001)

The SDBPS assesses general satisfaction/dissatisfaction with nine body parts (waist, thighs, hips, buttocks, legs, weight, figure, overall build, stomach) on a Likert scale with 1= *Extremely satisfied* and 5 = *Extremely dissatisfied* as anchors. The SDBPS has a univariate factor structure comprising all items and satisfactory validity among young Chinese women (e.g., Jackson and Chen, 2011, 2014; Chen and Jackson, 2012). In this study, the scale alpha was α = 0.87.

##### Positive and Negative Affect Schedule (PANAS; Watson et al., 1988)

The PANAS comprises 10-item positive affect and 10-item negative affect subscales and was used to obscure the Study 1 purpose and evaluate condition differences in pre-exposure affect. Women rated how they felt during the past 4 weeks regarding each PANAS adjective using a scale from “1 = *none or little of the time*” to “4 = *most of the time*.” Except for one excluded item (“alert”), the original PANAS structure was replicated in Chinese samples (Jackson and Chen, 2008b). Alphas were satisfactory for positive affect (α = 0.80) and negative affect (α = 0.82).

#### Design and Data Analysis

Chi-square tests and univariate ANOVAs assessed condition differences on age, relationship status and duration, BMI, affect, trait body dissatisfaction, pre-test and post-exposure state body dissatisfaction. Effects of image type on changes in state body dissatisfaction were assessed in a 2 (phase: pre-test vs. post-exposure to image) × 3 (presentation group: thin A4 challenge vs. thin control vs. average size control) repeated measures ANOVA. G^*^Power was used to generate sample size estimates based on a power analysis of the analysis. On the basis of assessing three groups and two measurements of the DV, using an alpha of 0.05, a power of 0.80, an effect size of 0.25, and a correlation between repeated measures of *r* = 0.85, the estimated sample size was *N* = 186.

Hierarchical multiple regression analyses were run within each condition to assess the impact of pre-test trait body dissatisfaction and BMI on changes in state body dissatisfaction. Post-exposure state body dissatisfaction was the criterion variable. Pre-test state body satisfaction was entered in Block 1 followed by trait body dissatisfaction (or BMI) in Block 2 of each model. With this approach, the impact of trait body dissatisfaction on changes in pre- to post-exposure state body dissatisfaction could be evaluated, independent of pre-test state—trait body dissatisfaction correlations. In line with the preceding power analysis estimate Group N's of 60 to 66 were considered sufficient for these analyses based on Tabachnick and Fidell (2007).

### Results

#### Manipulation Checks

There were no errors in identifying captions that accompanied male filler images, suggesting that general attention to the task was adequate and did not differ between conditions. Two women each from A4 challenge and thin non-A4 challenge conditions guessed the study was designed to assess how images affect their reactions to physical appearance. Responses of these women were retained because none of them linked their reactions to image presentations with their baseline (Study 1) “state” reactions and groups did not differ in their appraisals of appearance satisfaction as the hypothesis focus, $\chi_{\left(2\right)}^{2}$ = 2.01, *p* = 0.364.

#### Preliminary Analyses

Presentation groups did not differ on relationship status, $\chi_{\left(2\right)}^{2}$ = 0.33, *p* = 0.849. Table 4 summarizes descriptive statistics and univariate F-values for other measures. No significant group differences were found.

**Table 4:** Image presentation condition differences on research measures (*N* = 205).

	**Image presentation condition**				
	**Thin A4 challenge (*n* = 70)**	**Thin non-A4 challenge (*n* = 63)**	**Average size control (*n* = 72)**		
**Measure**	**Mean (*SD*)**	**Mean (*SD*)**	**Mean (*SD*)**	***F***	**Partial Eta^**2**^**
Age (years)	20.24 (1.61)	19.90 (1.51)	20.19 (1.71)	0.84	0.01
Relationship duration (weeks)	5.57 (12.58)	3.84 (7.81)	4.36 (10.96)	0.46	0.00
Body mass index	20.31 (2.38)	20.51 (2.24)	20.37 (2.60)	0.12	0.00
Trait body dissatisfaction	20.46 (5.75)	20.37 (5.79)	19.61 (6.02)	0.43	0.00
Positive affect	25.69 (4.57)	25.51 (4.07)	25.31 (4.41)	0.14	0.00
Negative affect	15.20 (3.68)	16.44 (3.89)	15.47 (3.65)	1.97	0.02
State body dissatisfaction (pre-test)	7.09 (5.54)	7.17 (5.18)	6.33 (5.55)	0.50	0.01
State body dissatisfaction (post-exposure)	7.04 (5.02)	7.35 (5.07)	6.67 (5.07)	0.30	0.00

*All univariate F-values were not significant (p's > 0.05)*.

#### Main Analyses

##### Effects of Image Presentations on State Body Dissatisfaction

In the ANOVA for changes in state body dissatisfaction, main effects of Phase, *F*_(1,202)_ = 0.87, *p* = 0.353, Condition, *F*_(2,202)_ = 0.41, *p* = 0.666, and their interaction, *F*_(2,202)_ = 0.45, *p* = 0.641, were not significant. On average, women who viewed images of peers passing the A4 challenge and commenting on this success did not report an exacerbation in state body dissatisfaction compared to women in either control condition.

##### Trait Body Dissatisfaction as a Predictor of Changes in State Body Dissatisfaction

Table 5 presents hierarchical regression analysis results. Within each condition, pre-exposure state dissatisfaction had a very high correlation with post-test state body dissatisfaction, explaining over *R*^2^ = 0.80 of the model variance. After controlling for pre-test state body dissatisfaction, elevations in pre-test trait body dissatisfaction predicted increases in post-exposure state dissatisfaction among women who viewed A4 challenge images, β = 18, *R*^2^ = 0.011, *p* = 0.04, but not women from other groups (Table 4). When BMI replaced trait body dissatisfaction in prediction models, variance explained was trivial for exposure to A4 challenge images, *R*^2^ = 0.004, *p* = 0.517, thin control images, *R*^2^ = 0.000, *p* = 0.972, and average size control images, *R*^2^ = 0.001, *p* = 0.859.

**Table 5:** The impact of trait body dissatisfaction on changes in state body dissatisfaction within conditions of female image exposure experiment (*N* = 205).

		**Experimental condition [female appearance image category viewed]**								
		**Thin A4 challenge**			**Thin non-A4 challenge**			**Average size non-A4 challenge**		
**Block**	**Predictor**	**β**	***pr***	***t***	**β**	***pr***	***t***	**β**	***pr***	***t***
1	State body dissatisfaction (pre-test)	0.90	0.90	17.23^**^	0.90	0.90	15.80^**^	0.90	0.90	17.74^**^
	Unique impact of Block 1 measure	R^2^ change = 0.814^**^ F change (1, 68) = 297.01^**^			R^2^ change = 0.804^**^ F change (1, 61) = 249.73^**^			R^2^ change = 0.818^**^ F change (1, 70) = 314.66^**^		
2	Trait body dissatisfaction (pre-test)	0.18	0.25	2.09^*^	0.09	0.12	0.93	0.07	0.08	0.67
	Unique impact of Block 2 measure	R^2^ change = 0.011* F change (1, 67) = 4.39^*^			R^2^ change = 0.003 F change (1, 60) = 0.86			R^2^ change = 0.001 F change (1, 69) = 0.45		

* *p < 0.05;*

** *p < 0.001*.

### Discussion

Study 3 predictions were partially supported. Contrary to the initial hypothesis, women who viewed images of peers successfully completing the A4 challenge did not experience a significant *mean* increase in state body dissatisfaction compared to women who viewed control images. This finding was not entirely surprising. Despite evidence of significant increases in body dissatisfaction following exposure to thin ideal imagery in select studies, meta-analyses have concluded that average group differences in general samples are modest and often not significant (e.g., Ferguson, 2013; Hausenblas et al., 2013). In line with such conclusions, viewing several images of peers passing the A4 challenge had no impact, *in general*, on state body dissatisfaction among women in the A4 challenge condition relative to women in control conditions. As Frederick et al. (2017) have written, exposure to thin ideal imagery does not inevitably increase body dissatisfaction; for some, exposure may increase positive self-evaluations of appearance but others are largely unmoved by exposure.

Within condition analyses illuminated the possible impact of individual differences in trait body dissatisfaction and BMI on state body dissatisfaction following exposure to A4 challenge images vs. control images designed to de-emphasize model physical appearance. Among women exposed to successful A4 challenge portrayals reflecting the thin ideal, those who reported elevations in pre-test trait body dissatisfaction experienced significant exacerbation in post-exposure state body dissatisfaction, independent of their pre-test state body dissatisfaction levels. In contrast, trait body dissatisfaction did not predict significant changes in state body dissatisfaction among women in either control condition. This pattern converges with contentions that exposure to idealized images increases state body dissatisfaction and state body satisfaction, respectively, among women who are already unhappy (Ferguson, 2013) or satisfied (Frederick et al., 2017) with their body. The effect of interest was modest in magnitude but suggests between-conditions comparisons in future experimental studies should be supplemented with analyses exploring factors that affect within condition variability in reactions to more vs. less idealized images.

## General Discussion

In this research, we examined correlates and effects of the A4 challenge, a social media meme that has emerged in China, literally promoting a paper-thin waist as the picture of health. Given that thinness is also central to modern Chinese views of feminine beauty (Chen et al., 2006; Jung, 2018; Zhang et al., 2018), we expected the A4 challenge would have more appeal for women than men. Analyses of Study 1 gender differences supported this contention: young women, especially those with lower BMIs, and their female peers were more aware of what the challenge entailed and had taken the challenge themselves more often than young men had. On average, women also had more positive views than men did of using the A4 challenge as a general index of overall health. Even though WC has links to chronic disease risk and mortality in Chinese samples (e.g., Koster et al., 2008; Zhang et al., 2009), the A4 challenge provides only a crude proxy of WC and is unlikely to have much utility as a physical health index.

Nonetheless, Study 2 comparisons indicated Chinese women who pass the A4 challenge accrued several psychological benefits relative to average size women, including fewer weight concerns, reduced social pressure related to losing weight or changing physical appearance, and lower levels of self-recrimination when eating larger amounts of food, albeit advantages did not extend to appearance comparisons with peers. These differences dovetail with the premise that smaller discrepancies between current vs. ideal body size result in less body dissatisfaction in cultures that emphasize the thin feminine attractiveness ideal (Thompson and Stice, 2001).

Study 2 women who passed the A4 challenge also reported significantly fewer overall eating disorder symptoms than average weight women did; this effect was a function of the latter group endorsing less favorable views of their weight and more negative emotional reactions to binge-eating. In contrast, groups did not differ on items that tapped binge-eating behaviors or compensatory practices to stem effects of overeating. As such, reduced binge-eating and/or increased use of potentially damaging weight loss strategies, such as dieting and laxative use were unlikely to account for the thinner waists and lower BMIs of women who passed the A4 challenge. Health practices related to diet composition, physical activity and sleep affect WC and weight status, yet genetics should not be discounted. Research based on twins raised together and apart has estimated that 66% of the variance in WC among women is due to additive genetic effects and unique environmental effects account for remaining variance (Nelson et al., 1999).

Given such data, framing the A4 task as a “challenge” that is achievable through effort, willpower, or particular practices mischaracterizes and over-simplifies influences on WC. Hence, rather than disseminating a narrow attractiveness standard that ignores genetic factors and applies to few women, promotion of diverse conceptualizations of attractiveness in media and advertising may help to foster increased body acceptance among young Chinese women.

Average weight women from Study 2 experienced more weight concerns and appearance pressure than thinner peers who passed the A4 challenge did, yet Study 3 indicated mere exposure to A4 challenge portrayals vs. control images designed to de-emphasize physical appearance did not result in a significant mean group difference in state body dissatisfaction. This apparent discrepancy between Studies 2 and 3 suggests that, for young Chinese women *in general*, pressure experienced from appearance media is more salient to body dissatisfaction than is exposure *per se* to thin ideal imagery (e.g., Jackson et al., 2016; Cai et al., 2020). However, within conditions analyses of Study 3 women indicated those who reported higher pre-existing levels of trait body dissatisfaction experienced modest, significant exacerbations in state body dissatisfaction as a result of exposure to A4 challenge images; in contrast, trait body dissatisfied women exposed to control images did not report significant exacerbations on this outcome.

Past meta-analyses have found limited exposure to thin ideal portrayals perpetuates state body dissatisfaction levels among at-risk Western women exposed to mass media depictions of thinness (e.g., Ferguson, 2013; Hausenblas et al., 2013). Study 3 suggested that such effects may extend to trait body-dissatisfied young Chinese women exposed to peer depictions of thinness, albeit replications are needed to ensure this conclusion is reliable, given the small effect size observed for trait body dissatisfaction in this condition. Coupled with evidence of negative weight perceptions and appearance pressure among average weight women in Study 2, these results suggest media literacy, outreach, and/or social advocacy interventions to increase awareness of causes and costs of thin ideal pursuit should be tested as strategies to combat unrealistic media portrayals of attractiveness, nurture self-acceptance, and promote body-positive communities (e.g., Jackson and Chen, 2015; Luo et al., 2021).

Notwithstanding its implications, the main limitations of this research should be noted. First, although university-age Chinese women are at risk for weight and eating concerns (Chen and Jackson, 2008; Zhang et al., 2018), results may not apply to other age and education cohorts in China or women in other cultures. Second, due to its cross-sectional design, Study 2 could not illuminate whether body image differences between average weight women and women who pass the A4 challenge resulted from or increased risk for weight status and body size differences. Third, even though the causal impact of viewing A4 challenge images on changes in state body dissatisfaction was tested in Study 3, effects on other potentially relevant outcomes such as state affect and state satisfaction with specific body parts (e.g., thin waist, hips, stomach) are not known. On a related note, it is possible that unmeasured factors, such as socioeconomic status, sexual orientation, and self-esteem also influenced perceptions of or reactions to the A4 challenge. Extensions based on more varied samples, research designs, possible correlates, and outcome measures can address these limitations.

To summarize, Westernization and Western media have been emphasized in explanations of body image concerns in non-Western cultures including China, often without any appreciation of potentially relevant local cultural forces (Jackson et al., 2016, 2020). Toward redressing such omissions, this research explored reactions to the A4 challenge in Chinese young adult samples. Study 1 analyses of gender differences confirmed the increased salience this challenge has for young women compared to young men. Study 2 underscored how young women who pass the challenge experience fewer weight concerns and less appearance pressure than average weight peers do and how passing the challenge is not related to reduced binge-eating or increases in dieting or other weight loss behaviors. Study 3 failed to demonstrate A4 challenge images cause state body dissatisfaction increases for a majority of women who viewed them. However, among those who were already generally unhappy with their body, exposure to A4 challenge portrayals but not control group portrayals predicted greater risk for exacerbations.

In conclusion, interest in the A4 challenge has apparently waned since 2016. However, news stories from March 2021 highlighted two new thinness “challenges” that have emerged recently in Chinese social media. In the “Manhua waist challenge,” netizens try to mimic the awkward “head-down, elevated thin waist and buttocks” pose of an animated female character; the other thinness challenge features young adult women fitting into child size clothing. These trends underscore how thinness “challenges” continue to be perpetuated in Chinese social media, albeit the manner in which they are expressed appears to change over time. In light of our findings and the persistence of thinness challenge memes in Chinese social media, researchers in China and other non-Western nations should continue to explore how idiosyncratic local expressions of physical attractiveness ideals in social media affect their citizens.

## Data Availability Statement

The raw data supporting the conclusions of this article will be made available by the authors, without undue reservation.

## Ethics Statement

The studies involving human participants were reviewed and approved by Southwest University Human Research Ethics Committee. The patients/participants provided their written informed consent to participate in this study. Written informed consent was obtained from the individual(s) for the publication of any potentially identifiable images or data included in this article.

## Author Contributions

TJ contributed to the conceptualization of the research, data analyses, and wrote the final drafts of all paper sections. XY completed all the data collection and assisted in data analyses. BH contributed to the initial conceptualization of the research. HC contributed to the conceptualization of the research. All authors contributed to the article and approved the submitted version.

## Conflict of Interest

The authors declare that the research was conducted in the absence of any commercial or financial relationships that could be construed as a potential conflict of interest.
